# Dataset of dual RNA-sequencing of *Phytophthora palmivora* infecting coconut (*Cocos nucifera* L.)

**DOI:** 10.1016/j.dib.2020.105455

**Published:** 2020-03-18

**Authors:** K.P. Gangaraj, M.K. Rajesh

**Affiliations:** a ICAR-Central Plantation Crops Research Institute, Kasaragod 671124, Kerala, India; b Mangalore University, Mangalagangotri, Mangaluru 574199, Karnataka, India

**Keywords:** Dual RNA-seq, Transcriptomics, Coconut, *Phytophthora palmivora*, Gene Ontology

## Abstract

*Phytophthora* spp. is an oomycetes pathogen which causes serious damage to a wide range of crops. Bud rot disease of coconut palm, caused by *P. palmivora*, causes huge economic losses since it cannot be detected at an early stage. Utilizing dual RNA-sequencing (RNA-seq), we have simultaneously investigated the gene expression patterns in both, the infecting oomycete (*P. palmivora)* and infected host (coconut leaflets). Samples were collected at three time points viz., 12, 24 and 36 h, from both infected and uninfected (control) tissues and subjected to RNA-seq on an Illumina Hiseq™ 2500 sequencing platform. High quality reads obtained were subjected to mapping with corresponding reference genomes by using the HISAT2/ StringTie package. A total of 81,683 transcripts were generated against the coconut reference genome, while 9340 transcripts were generated against *P. palmivora* genome. Out of these, a total of 64,639 coconut transcripts and 9168 *P. palmivora* transcripts could be annotated using BLASTx. Gene ontology (GO) analysis, carried out using Blast2GO, resulted in 212,643 coconut and 30,736 *P palmivora* transcripts being functionally classified, with a single gene product described by numerous terms under the three classifications. The insights obtained could contribute to an understanding of pathogenesis of *P. palmivora* and inducible defense response of coconut leaves to *P. palmivora*.

Specifications table

**Table:** 

Subject	Biology
Specific subject area	Transcriptomics
Type of data	Dual RNA-sequencing (RNA-seq) data
How data were acquired	Illumina Hiseq™ 2500 sequencing platform
Data format	Raw sequencing data (fastq) and analyzed data (fasta)
Parameters for data collection	Dual RNA-seq of coconut-*Phytophthora* interactions at different time intervals
Description of data collection	We employed time-resolved dual-transcriptomic approach to decipher *Cocos nucifera*-*Phytophthora palmivora* interactions. This approach revealed different pathogen and host transcriptome dynamics.
Data source location	Kasaragod, India (12°32′38.0″N; 74°57′45.7″E).
Data accessibility	Repository name: NCBI SRA Data identification number: PRJNA544637 The dataset includes six records, from SRR9140949 to SRR9140954. Direct URL to data: https://www.ncbi.nlm.nih.gov/sra/PRJNA544637

## Value of the data

• The dual RNA-seq dataset provides the first resources for coconut–*P. palmivora* interaction studies and can be leveraged in deciphering the molecular mechanisms underlying response of coconut to *P. palmivora* attack.
• The data allows further analysis to identify candidate effector genes in *P. palmivora* that possibly contribute to manipulation of host processes and promote infection.
• This data could be useful for selection of disease resistant varieties for coconut breeding programs and design of novel disease management startegies.
• It is a useful reference transcriptome to other researchers working in palm diseases caused by *Phytophthora* spp.

## 1 Data

**Fig. 1:**
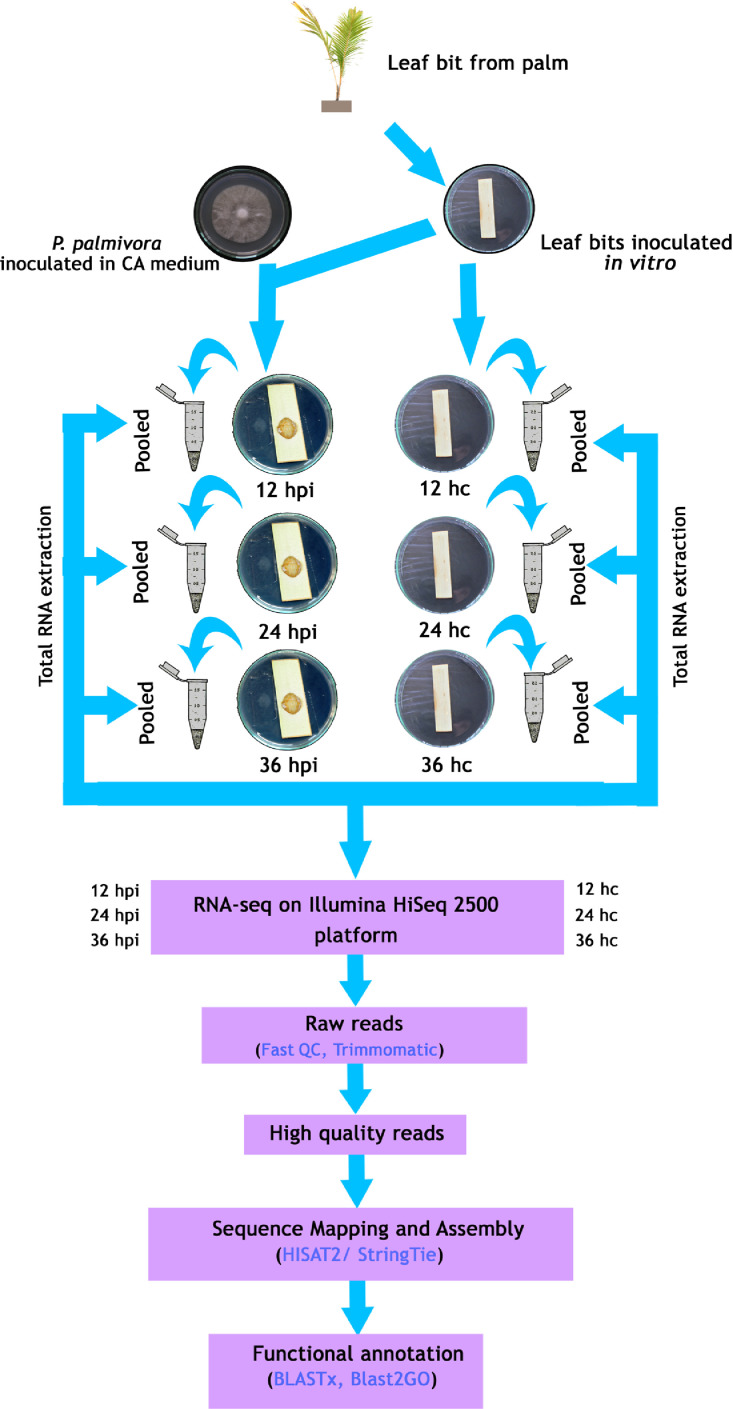
Schematic outline of the experimental design and data analysis workflow.

**Fig. 2:**
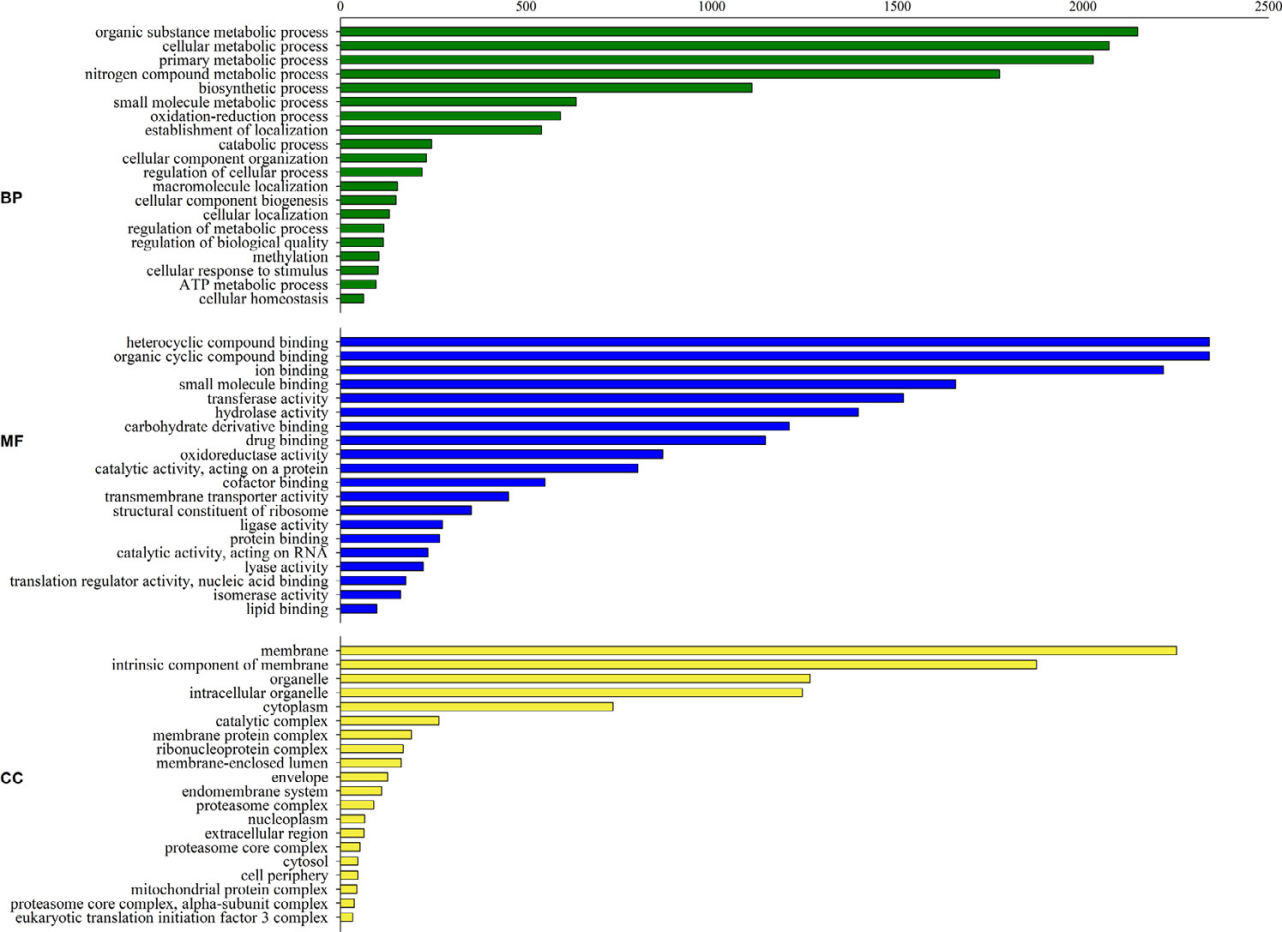
A combined graphical representation of Gene Ontology (GO) analysis of annotated coconut transcripts- biological process (BP), molecular function (MF), and cellular component (CC). Number of sequences can vary amongst the different classifications as a single gene product can be described by numerous terms in the three classifications.

**Fig. 3:**
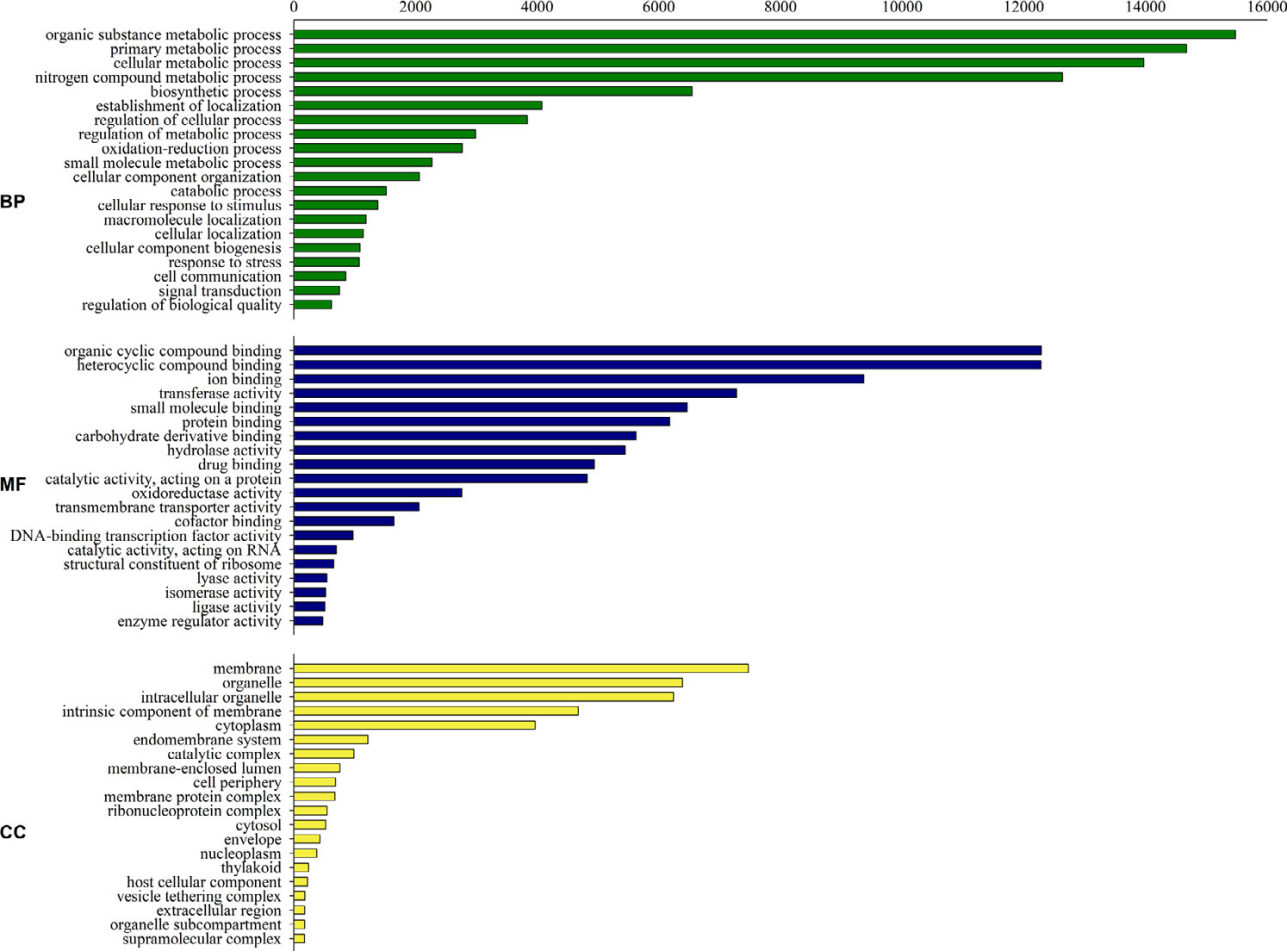
A combined graphical representation of Gene Ontology (GO) analysis of annotated *P. palmivora* transcripts- biological process (BP), molecular function (MF), and cellular component (CC). Number of sequences can vary amongst the different classifications as a single gene product can be described by numerous terms in the three classifications.

Schematic outline of the experimental design and RNA-seq data analysis workflow is given in Fig. 1. The details of RNA-seq data submitted to the NCBI Sequence Read Archive (SRA) are provided in Table 1. Table 2 gives an overview of the RNA-seq statistics of raw read and clean reads obtained at different time intervals. Mapping statistics of high quality reads with coconut genome is given in Table 3 and with *Phytophthora palmivora* genome is given in Table 4. Figs. 2 and 3 display the functional classification of coconut and *P. palmivora* in three Gene Ontology (GO) categories viz., biological processes, molecular functions and cellular components, respectively.

**Table 1:** Details of RNA-seq data submitted to the NCBI Sequence Read Archive (SRA).

Accession no.	Bioproject accession no.	Biosample accession no.	Library id
SRR9140951	PRJNA544637	SAMN11867028	12hc
SRR9140952	PRJNA544637	SAMN11867027	12hpi
SRR9140949	PRJNA544637	SAMN11867030	24hc
SRR9140950	PRJNA544637	SAMN11867029	24hpi
SRR9140953	PRJNA544637	SAMN11867032	36hc
SRR9140954	PRJNA544637	SAMN11867031	36hpi

[12hc: 12 h control, 12hpi: 12 h post inoculation; 24hc: 24 h control, 24hpi: 24 h post inoculation; 36hc: 36 h control, 36 hpi: 36 h post inoculation].

**Table 2:** RNA-seq statistics of raw read and clean reads obtained at different time intervals.

Sample name	Number of paired end reads (raw reads)	GC%	Read length (bp)	Number of paired end reads (clean reads)
12hc	64,921,052	47.88	100 × 2	64,503,262
12hpi	63, 662,338	47.85	100 × 2	63,090,348
24hc	63,064,844	47.74	100 × 2	62,601,136
24hpi	67,291,090	48.53	100 × 2	66,770,394
36hc	70,446,814	48.02	100 × 2	70,150,788

**Table 3:** Summary of mapping information of pre-processed reads against coconut genome.

Sample name	Number of reads considered for alignment	Total number of reads aligned	Number of unaligned reads
12hc	64,503,262	62,764,100	3041,832
12hpi	63,090,348	61,559,938	2583,208
24hc	62,601,136	60,771,365	3271,750
24hpi	66,770,394	63,925,486	4296,428
36hc	70,150,788	67,896,254	3820,556
36hpi	60,135,364	57,801,520	3500,526

**Table 4:** Summary of mapping information of unaligned reads against *P. palmivora* genome.

Sample name	Total number of reads considered for alignment	Total number of reads aligned	Total number of unaligned reads
12hc	3041,832	1009	3041,784
12hpi	2583,208	1499	2583,056
24hc	3271,750	1235	3271,696
24hpi	4296,428	855,123	3475,146
36hc	3820,556	1077	3820,346
36hpi	3500,526	569,557	2957,968

A total of 81,683 transcripts were generated against the coconut reference genome, while 9340 transcripts were generated against *P. palmivora* genome. A total of 64,639 coconut transcripts and 9168 *P. palmivora* transcripts could be annotated using BLASTx program. Gene Ontology (GO) analysis, performed to assign GO identity to the annotated transcripts, revealed that 212,643 coconut transcripts could be assigned with GO terms (Biological processes = 90,956; Molecular functions = 85,563; Cellular components = 36,124) (Fig. 2). Among the *P. palmivora* transcripts, 30,736 sequences were assigned with GO terms (Biological processes = 12,634; Molecular functions = 11,706; Cellular components = 6396) (Fig. 3). We provide the first molecular resource of the interactions occurring between coconut and *P. palmivora by* simultaneously analysing the global gene expression of the oomycete and host by dual RNA-seq.

## 2 Experimental design, materials, and methods

### 2.1 Experimental design and sampling

The *in vitro* inoculation assay, standardized earlier in our laboratory [1], was utilized for the infection studies. Briefly, spindle leafles from two year old coconut seedlings [Chowghat Orange Dwarf (COD) cultivar] were taken and inoculated in plain Y3 media [2] in Petri plates. Zoospore suspension (10 µl of 2 × 10^4^ mL^−1^) of a virulent isolate of *P. palmivora* (GenBank accession no. MK500842.1), isolated from bud rot infected coconut palm in our laboratory and maintained in carrot agar (CA) medium, was used for the inoculation experiments. *P. palmivora* was inoculated onto pin pricked coconut leaflets. Typical necrotic lesions, indicating infection, were first observed after 12 h post inoculation (hpi). Samples were taken at 12 hpi, 24 hpi and 36 hpi. Un-inoculated, but pin pricked leaflets, served as controls (hc). Three biological replicates were sampled at each time point and used for isolation of RNA.

To confirm the presence of the pathogen at the infection area, the infected area was wiped with alcohol, DNA was extracted from the specific area using DNeasy Plant Mini Kit (Qiagen, USA) and amplified using *Phytophthora*-specific primers (ITS6 and ITS4) [3]. An expected band of around ∼900 bp was obtained, which was sequenced. Analysis of the sequence by BLASTn revealed complete identity to *P. palmivora* (GenBank accession no. GU111653).

### 2.2 RNA extraction and sequencing

Total RNA was extracted from 150 mg of tissue at each specific time points [from the infection area in case of inoculated samples and pricked area in the case of control samples] using the NucleoSpin^Ⓡ^ RNA Plant Kit (Macherey-Nagel). Total RNA was extracted from three independent samples per treatment. The quality and the purity of the extracted RNA were assessed by OD 260 nm/280 nm ratio and RNA integrity number was analyzed using an Agilent Technologies 2100 Bioanalyzer with the Agilent RNA chip with RIN (RNA Integrity Number) > 8.0. The isolated RNA samples, from each particular satge, were pooled together and used for RNA-seq library construction (TruSeq RNA Sample Prep Kit, Illumina). After quality assessment, the constructed messenger RNA libraries were subjected to paired-end sequencing on an Illumina HiSeq 2500 platform, as per the procdure described in Rajesh et al. [4].

### 2.3 Data analysis

Raw reads, quality headed (.fastq) fasta files were subjected to quality check. Initially, the raw reads were checked for the ambiguous bases, Phred score >Q20, read length, nucleotide base content and other parameters using FastQC [5]. Trimmomatic was then used to filter low-quality sequences [6]. After trimming and filtering of the low quality reads, a QC was performed in order to reassess the quality of reads. Mapping and assembly of the good quality reads to the reference genomes was performed with ‘New Tuxedo Suit’ (HISAT2/StringTie), using default parameters [7]. The reference genomes of coconut (PRJNA374600; Xiao et al. [8]) and *Phytophthora palmivora* (PRJNA318026; [9]) were downloaded from NCBI. Initially, indexing of the coconut reference genome and alignment of reads to the coconut reference genome was done using HISAT2. The aligned reads are then taken and used for the transcript assembly using the StringTie Tool. The unaligned reads were aligned with *P. palmivora* genome.

### 2.4 Transcriptome functional annotation

Local protein databases were created from five organisms viz., Phytophthora palmivora, *Phytophthora megakarya* (PRJNA318028; [9]), Hainan Tall coconut (PRJNA374600; [8]), date palm (PRJNA249070; [10]) and oil palm (PRJNA268357; [11]). The assembled transcriptomes, of both host and the pathogen was annotated by BLASTx search against corresponding proteome databases, keeping a threshold of 1 × 10^−4^ maximum e value. Blast2GO [12] were used to perform Gene Ontology (GO) analysis of the assembled transcriptome and individually for *P. palmivora* and coconut.

## Acknowledgements

The authors would like to thank Indian Council of Agricultural Research (ICAR) and Department of Biotechnology, Government of India (Grant number: BT/BI/04/053/2002) for funding (Distributed Information sub-center).

## Conflict of Interest

The authors declare that they have no known competing financial interests or personal relationships that could have appeared to influence the work reported in this paper.

## CRediT authorship contribution statement

**K.P. Gangaraj:** Data curation, Writing - original draft. **M.K. Rajesh:** Conceptualization, Methodology, Supervision, Writing - review & editing.

## Appendix Supplementary materials

Image, application 1
